# Mechanical Properties of Surface-Treated Bamboo Strip-Reinforced Biobased Polyamide Composites

**DOI:** 10.3390/polym17101379

**Published:** 2025-05-17

**Authors:** Clément Pébère, Gautier Mangeret, Eric Dantras, Colette Lacabanne, Jany Dandurand, Thomas Moussiegt, Edouard Sherwood, Gilles Hochstetter

**Affiliations:** 1 CIRIMAT, Université Toulouse 3—Paul Sabatier 118 Route de Narbonne, 31062 Toulouse, France; clement.pebere@univ-tlse3.fr (C.P.); gautier.mangeret@univ-tlse3.fr (G.M.); eric.dantras@univ-tlse3.fr (E.D.); jany.lods@univ-tlse3.fr (J.D.); 2 Cobratex, 1 Allée de l’Orchidée, Local 8-ZA Activestre, 31390 Carbonne, France; thomas.moussiegt@cobratex.com (T.M.); edouard.sherwood@cobratex.com (E.S.); 3 Arkema, 420 Rue d’Estienne d’Orves, 92700 Colombes, France; gilles.hochstetter@arkema.com

**Keywords:** bamboo strips, fire-retardant polyamide 11, bamboo thermal properties, mercerization treatment, dynamic mechanical analysis, shear modulus

## Abstract

Fully bio-based composites were obtained from continuous bamboo strips and flame-retardant polyamide 11 (PA11-FR) matrix. A mercerization treatment was performed on the bamboo strips surface to optimize fiber-matrix interactions. Composites were obtained by thermocompression molding with two pressure plateaus. The influence of the concentration of NaOH solution treatment was analyzed. The thermogravimetric analysis highlighted that the mercerization treatment removes part of hemicellulose, low molecular weight lignin and amorphous cellulose, while crystalline cellulose is preserved. Dynamic mechanical analysis performed in the shear configuration revealed the level of interactions between bamboo strips and PA11-FR matrix. The glassy modulus was improved for the composites compared to the matrix and their rubbery modulus was increased by a factor 4.6. Composites with bamboo strips treated at 1% NaOH showed the highest shear modulus across the entire temperature range with an increase by a factor of 1.39 on the glassy plateau and 1.3 on the rubbery plateau, with the untreated bamboo strips/polyamide 11-FR composite as reference. Water uptake was analogous for composites and bamboo strips, so the shear modulus at room temperature was not impacted by moisture.

## 1. Introduction

Future environmental issues promote the development of new materials for an industry more sensitive to its ecological footprint [1]. The use of natural compounds has shown a growing potential over the past decades in the field of composite materials [2,3]. Among natural reinforcement materials, plant fibers exhibit interest due to their abundance, renewable characteristics and mechanical performances [4,5]. Sisal, hemp, flax and bamboo have been widely studied to be used as a reinforcement for polymer-based composites [6,7]. Bamboo has revealed multiple reasons to be used as a reinforcement: mechanical behavior, Young’s modulus, fast maturity cycle (3–4 years) and ubiquitous properties [8,9,10]. A previous study on Phyllostachys Viridiglaucescens (bamboo species) showed that thermal stability and mechanical performances are not related to native regions and species. However, the proportion of crystalline cellulose is more important in the outer part of bamboo culm [11,12].

To propose fully biobased composites, organic thermoplastic matrices have shown a growing interest [13]. To match the degradation temperature of natural fibers starting at 200 °C, a polymer with a long aliphatic sequence is required. Accordingly, polyamide 11, synthesized from castor oil, is a semi-crystalline polymer with a low melting temperature (187 °C) [14,15]. Flame certification is one of the aeronautics standards which could limit the use of biobased polymers. In order to pass this certification, flame-retardant particles have been added to the polymer matrix [15,16,17,18].

Thermoplastic composites reinforced by bamboo fibers have been widely studied, and one of the main drawbacks is the shaping of these fibers for processing. Bamboo strips are another shape of bamboo reinforcement, which can be easier to shape in an industrial process compared to fibers [11,19,20]. However, the nonpolar surface of bamboo strips requires a surface treatment with the aim of improving interactions between the matrix and reinforcement material [21].

Alkaline treatment, also called mercerization [21,22], is the most common treatment realized on natural fibers and in particular on bamboo fibers for composite applications [23,24,25,26,27,28,29,30,31]. The effects of time, temperature and concentration have been studied on different natural fibers. According to Wu et al. [32], temperature is the parameter which impacts the most the crystallinity and the chemical composition of bamboo fibers. Previous researches demonstrate that mercerization treatment improves surface interactions between bamboo fibers and polymer matrix [33,34].

In the area of fully bio-based composites, the challenge is to fit the requirements of technical applications involving mechanical performances. This implies the use of continuous reinforcement with new morphologies like strips, allowing both flexibility and easier processing of composites. The treatment of such strips is an important issue for improving the interface with the matrix. This paper presents the effect of the concentration of mercerization treatment on the thermo-mechanical performances of bamboo strips and bamboo strip-reinforced PA11-FR composite. The evolution of bamboo strips was characterized by thermogravimetric analysis (TGA) and tensile tests. The influence of mercerization on the mechanical properties of bamboo strips/PA11-FR composites was assessed by dynamic mechanical analysis (DMA).

## 2. Materials and Methods

### 2.1. Materials

#### 2.1.1. Bamboo Strips

The bamboo species selected for this study was Phyllostachys Viridiglaucescens. Bamboo strips with dimensions of 10 mm in width and 150 mm in length were supplied by Cobratex (Carbone/France). Two groups of bamboo strips (BS) were selected to investigate Young’s modulus and ultimate strength according to the radial position in the culm. The first ones (EXT-BS) were extracted between 0 and 400 µm of the exterior of the bamboo lamella, and the second ones (MID-BS) were extracted between 400 and 800 µm of the exterior of the bamboo lamella.

#### 2.1.2. PA11-FR Matrix

PA11-FR is a bio-based flame-retardant polyamide 11 referred as Rilsan^®^ MB3000 NAT and supplied by Arkema (Serquigny/France) as a film with a thickness of 0.125 mm. The flame-retardant filler was melamine cyanurate (MC), as shown in a previous study [34].

#### 2.1.3. Mercerization Treatment

This study is focused on the NaOH concentration effects of the mercerization treatment. Temperature and time were selected according to the literature [32]. Three solutions were realized by adding NaOH in deionized water to reach concentrations of 1 wt%, 3 wt% and 5 wt%, respectively. Bamboo strips were immersed for 4 h at 50 °C. Then, they were washed with deionized water and dried for 6 h at 60 °C [23]. Samples were designated as UT, T1, T3 and T5 for bamboo strips without treatment (UT) and treated with the solution at 1 wt% NaOH (T1), 3 wt% NaOH (T3) and 5 wt% NaOH (T5), respectively.

#### 2.1.4. Composite Processing

**Figure 1:**
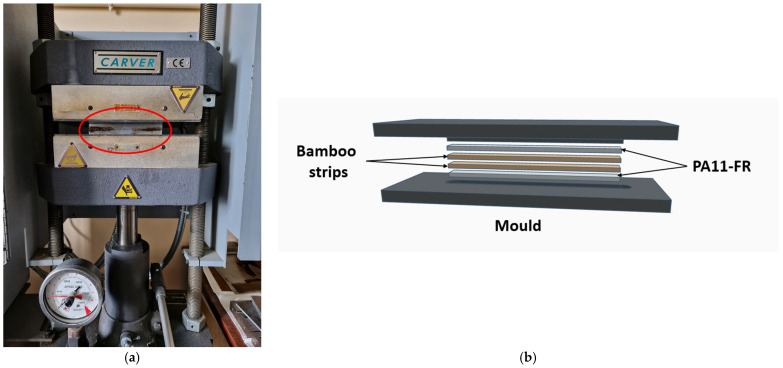
(**a**) Experimental set up for compression molding of composites and (**b**) 3D view of the mold for film stacking composite.

Composites were obtained by thermocompression molding. Figure 1 shows the Carver TM Bench Top Presses used and a 3D view of the film staking realized in the mold for composite processing. PA11-FR matrix and bamboo strips were cut into 10 × 100 mm^2^ rectangles. Two or three bamboo strips were placed between two polyamide films in order to respect a mass ratio of 65 ± 5%.

**Figure 2:**
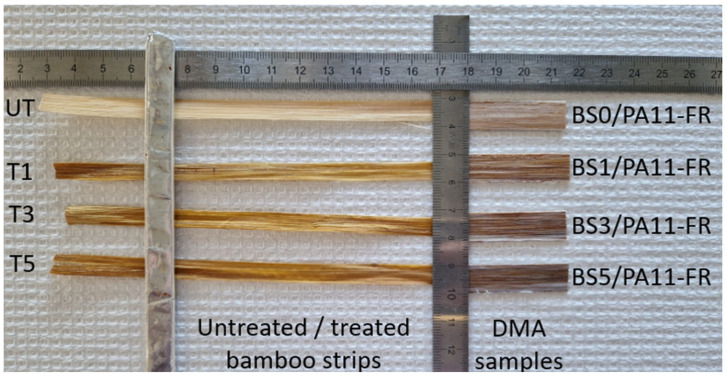
Untreated bamboo strips (UT) received from Cobratex, treated bamboo strips (T1, T3, T5), DMA samples of untreated bamboo strips/PA11FR composite (BS0/PA11FR) and treated bamboo strips/PA11FR composites (BS1/PA11FR, BS3/PA11FR, BS5/PA11FR).

The press was heated at 190 °C. The mold containing PA11-FR films and bamboo strips was placed on the lower heating plate. Plates were brought closer until the top of the mold was in contact with the upper plate for 90 s. Then, a low pressure (17 bars) was applied for 90 s and 68 bars were applied for 3 min. Finally, composites were naturally cooled at an estimated speed of 30 °C/min without any pressure. Samples were designated as BSX/PA11-FR, with X corresponding to the alkaline treatment of bamboo strips (1, 3 or 5 wt%). Composites obtained with bamboo strips without treatment were designated as BS0/PA11-FR. Using the same protocol with PA11-FR films, matrix samples with an equivalent thickness were made for the purpose of reference. Figure 2 shows bamboo strips used for composites processing and their composites obtained for DMA tests.

### 2.2. Methods

#### 2.2.1. ThermoGravimetric Analysis (TGA)

Analysis was performed on a TGA Q50 analyzer from TA Instrument (New Castle, DE, USA). ASTM standards were considered for measurement procedure (ASTM E2550-21) [35]. The weight of the samples was between 7 and 8 mg, and three samples were tested for the purpose of repeatability. Measurements were done under a passive atmosphere (nitrogen) and an oxidative one (synthetic air), with a temperature ramp of 10 °C/min from ambient to 800 °C.

#### 2.2.2. Tensile Test

Tensile tests were performed with the MTS Criterion model 43 electromechanical load frame from MTS System Corporation. Bamboo strips had a length of 15 mm, and the speed was 1 mm/min. At least 15 samples of each bamboo strip were tested. The value of Young’s modulus was extracted from the linear elastic region and the ultimate strength value was recorded at the maximum of the curve. Samples were referenced as UT-EXT and UT-MID, for bamboo strips without treatment coming from the layer 0–400 µm (EXT) and the layer 400–800 µm (MID), respectively. T1-EXT and T1-MID refer to bamboo strips treated with the solution at 1% NaOH coming from the layer 0–400 µm (EXT) and the layer 400–800 µm (MID), respectively.

#### 2.2.3. Dynamic Mechanical Analysis (DMA)

In this work, dynamic mechanical analysis was used to evaluate the mechanical properties of bamboo strips and their composites. Trials were performed in rectangular torsion mode on an ARES G2 strain-controlled rheometer produced by TA Instruments. The complex shear modulus $G^{*}\left(\omega ,T\right)$ was obtained as a function of temperature *T* and angular frequency ω

(1) $$G^{*}\left(\omega ,T\right)=G^{\prime }\left(\omega ,T\right)+iG^{\prime \prime }(\omega ,T)$$

where *G′* is the storage modulus and *G″* is the dissipative modulus. Measurements were performed at an angular frequency of 1 rad/s under a heating rate of 3 °C/min between −120 °C and 140 °C. In order to stay in the linear domain, a strain of 0.1% was applied. Two successive sweeps were performed to characterize the hydrated and anhydrous states of samples. Three samples of each were tested to address repeatability concerns. Samples dimensions were 40 mm in length, 10 mm in width, with a thickness of 0.648 ± 0.094 mm. Storage modulus G′ and dissipative modulus G″ were measured under conditions of the ASTM standard ASTM D7028-07(2015) [36].

#### 2.2.4. Water Uptake

Sodium nitrate (NaNO_2_) was chosen to simulate an atmosphere at 65% of relative humidity at 25 °C. This percentage of relative humidity corresponds to the standard of the aeronautic industry [37]. Each sample was put in an oven for 2 h at 150 °C and then weighted to define the reference weight of the sample without water. Then, the samples were placed in the atmosphere until their weight stabilized, in order to obtain hydrated samples. After the DMA tests, the weight was measured directly after the second ramp in order to determine water uptake.

## 3. Results

### 3.1. Thermal Stability

**Figure 3:**
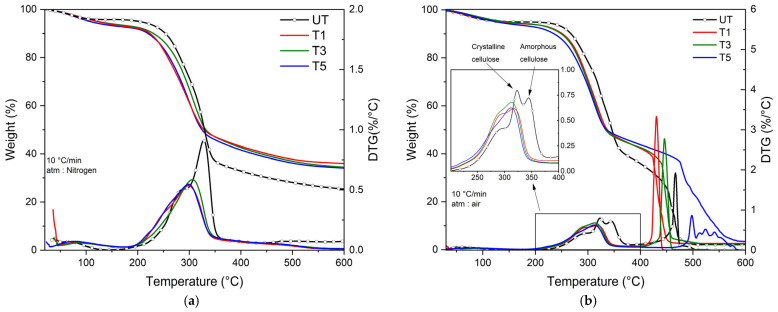
TGA and DTG thermograms of untreated and treated bamboo strips under nitrogen (**a**) and under air (**b**).

Thermal stability is one of the main parameters for bamboo strip processing. TGA allows us to investigate the effect of the mercerization treatment on thermal stability. Figure 3 shows TGA and DTG thermograms of untreated and treated bamboo strips under nitrogen (a) and synthetic air (b).

Under nitrogen (Figure 3a), the first event observed around 100 °C is water loss. The second is due to the decomposition of two major components of natural fibers: hemicellulose and cellulose. The residue is due to lignin i.e., the third main component of natural fibers. Water uptake for treated bamboo strips increases from 5 to 7 wt%. The amount of lignin is more important for treated bamboo strips than untreated ones. This could be explained by the degradation temperature of lignin, which is between 160 °C and 800 °C. The difference in lignin degradation temperatures is linked to its molecular weight: the higher the molecular weight is, the higher the temperature of degradation will be. The increase in lignin content reflects a stabilization in temperature of high-molecular-weight lignin [11,14,23,28].

Under air (Figure 3b), the presence of oxygen permits the degradation of lignin by the activation of the oxidization mechanism. DTG thermogram of untreated bamboo strips is different compared to the one recorded under nitrogen. Cellulose degradation DTG peak exhibits a new contribution at 345 °C, which is only visible for untreated bamboo strips. According to Song et al. [38], the lower the molecular weight of cellulose is, the more cellulose tends to crystallize. Accordingly, crystalline cellulose degrades before the amorphous. Generally, mercerization treatment allows us to remove part of lignin, hemicellulose and part of cellulose. We observe that the mercerization treatment permits the removal of hemicellulose, part of lignin and amorphous cellulose, while preserving crystalline cellulose, which is essential for maintaining the mechanical properties of natural fibers.

### 3.2. Tensile Properties

**Figure 4:**
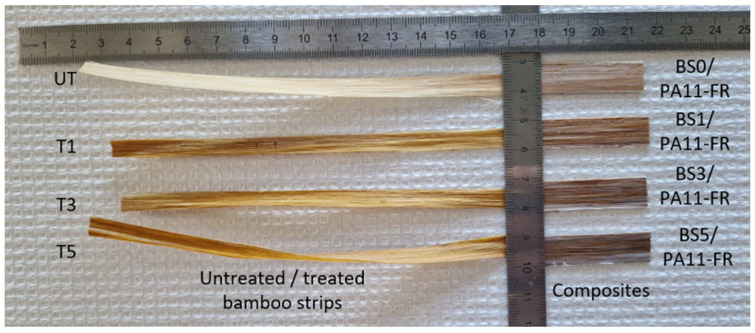
Bamboo strips before and after mercerization treatment and their composites.

Tensile tests need to be carried out on multiple samples to take into account the high variability of natural fibers. Figure 4 shows untreated bamboo strips, treated bamboo strips and their composites. It can be seen in Figure 4 that bamboo strips treated with 3 wt% NaOH (T3) and 5 wt% NaOH (T5) are twisted. Accordingly, only untreated bamboo strips (UT-BS) and 1 wt% NaOH treated bamboo strips (T1-BS) will be studied.

**Figure 5:**
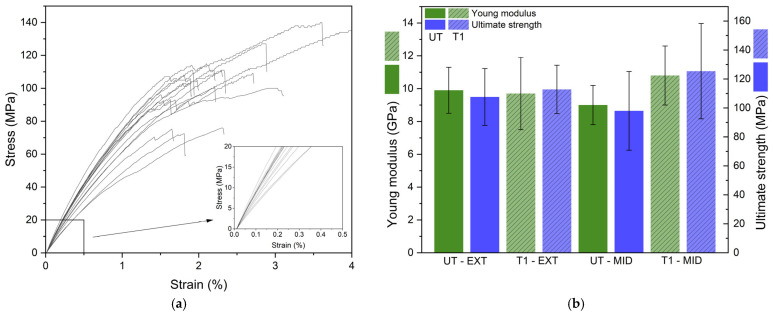
(**a**) Stress–strain curves of UT-EXT bamboo strips; (**b**) Young modulus and ultimate strength of UT-EXT, UT-MID, T1-EXT and T1-MID bamboo strips.

Figure 5a shows typical stress–strain curves of bamboo strips and data are reported on the histogram of Figure 5b, where the values of the Young modulus and ultimate strength of untreated bamboo strips (UT) and bamboo strips treated with 1 wt% NaOH solution (T1) are reported. Two series of lamella have been studied—EXT-BS and MID-BS—in order to analyses the effect of extraction area on bamboo strips. All values are reported in Table 1 [11,12,15,20,23,28].

Young modulus and ultimate strength of EXT-BS do not change upon mercerization treatment at 1% NaOH. For the MID-BS, variations of the average values of the Young modulus and ultimate strength are not significant. Taking error bars into consideration, both EXT-BS and MID-BS may be used for processing technical composites. For composite processing and analysis, only MID-BS are used. These bamboo strips will further be referred to as UT-BS.

### 3.3. Dynamic Mechanical Properties

**Figure 6:**
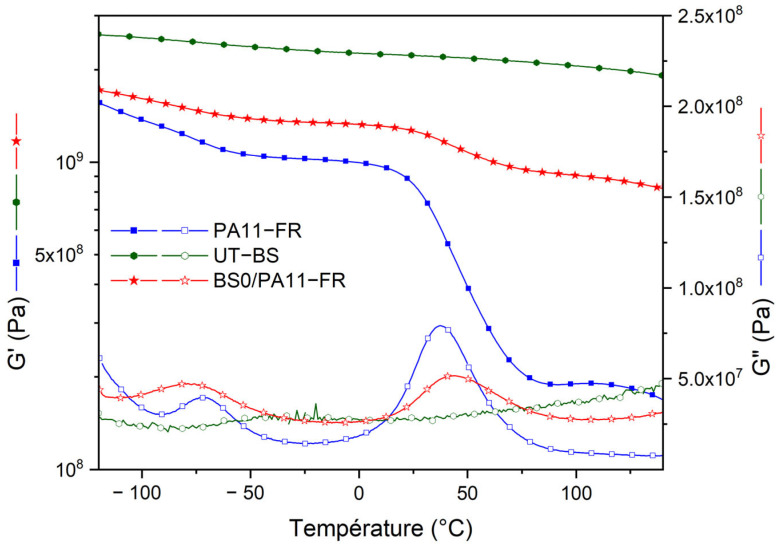
DMA thermograms of storage modulus G′ and dissipative modulus G″. Green line: untreated bamboo strip (UT-BS), red line: PA11-FR matrix; blue line: composites with untreated bamboo strips (BS0/PA11FR).

Figure 6 shows the second temperature ramp of the dynamic mechanical analysis thermograms for untreated bamboo strips (UT-BS), the polyamide 11 matrix (PA11-FR) and PA11-FR/untreated bamboo strips (BS0/PA11-FR) composites. Filled-symbol curves represent the conservative modulus (G′), while open-symbol curves represent the dissipative modulus (G″).

#### 3.3.1. Bamboo Strips

Bamboo strips do not show any significant variation of the storage and dissipative modulus in the explored temperature range. This is consistent with previous data reported on bamboo strips, showing that the only event having a significant effect on the conservative modulus is the α relaxation of hemicellulose, which starts at 160 °C [11]. Accordingly, the observation of a plateau for the G′ conservative modulus (G_g_ = 2.22 GPa at room temperature) in the observed temperature range is consistent with previous data.

#### 3.3.2. PA11-FR Matrix

The DMA thermogram of the PA11-FR matrix shows two plateaus on the storage modulus: a glassy plateau that ends at ≅0 °C with a shear modulus value of 1.02 GPa at −20 °C and a rubbery one that starts at ≅75 °C with a shear modulus value of 0.19 GPa at 100 °C. The intermediate region corresponds to the viscoelastic transition of the polymer. This transition is responsible for the dynamic loss peak designated as the α mode; it is located at T_α_ = 37 °C. At lower temperature, a less intense relaxation mode, designated as the β mode, is observed at T_β_ = −73 °C. According to Lods et al. [15], it has been associated with the localized mobility of amide groups of the PA11-FR polyamide.

#### 3.3.3. Untreated Bamboo Strips/PA11-FR Composite

The storage modulus of the untreated bamboo strips/PA11-FR composite is intermediate between the one of the matrix and the one of bamboo strips in the entire temperature range. Bamboo strips improve the shear modulus properties of the biobased polyamide, which was unexpected. The increase in the shear modulus on the glassy state is 28% (1.02 GPa → 1.31 GPa) and 363% on the rubbery plateau (0.19 GPa → 0.88 GPa). The viscoelastic domain is reduced upon the introduction of bamboo strips [33,34].

Moreover, it is observed that bamboo strips do not have any relaxation mode in this temperature range. Composites realized with untreated bamboo fibers exhibit two relaxation modes similar to those of the matrix. The β mode of the composite is larger and it exhibits a higher magnitude. This evolution suggests that amide–water interactions are more frequent in composites, which is consistent with the introduction of a natural reinforcement. The α mode of the composite is less intense compared to the one of the matrix according to the composition of composites. The introduction of polar sites with different physical interactions could explain the temperature shift of the maximum of the peak [27]: T_α,C_ = 41 °C for the composite, and T_α,M_ = 37 °C for the matrix.

#### 3.3.4. Treated Bamboo Strips/PA11-FR Composite

**Figure 7:**
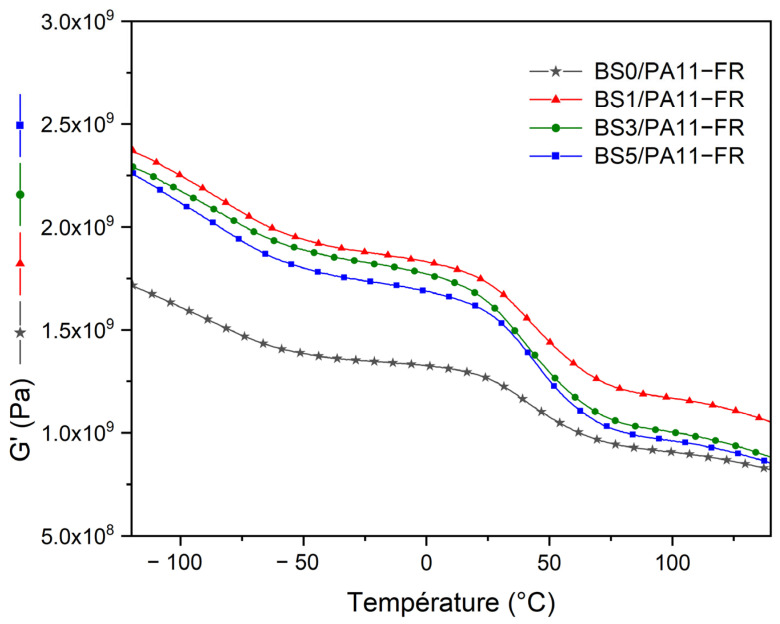
Storage modulus G′ of bamboo strips/PA11-FR composites with NaOH-treated bamboo strips at different concentrations.

Figure 7 shows the storage shear modulus of bamboo strips/PA11-FR composites, with bamboo strips treated with alkaline solutions at different concentrations. Composites with treated bamboo strips have a higher storage modulus compared to those with untreated bamboo strips.

In the entire temperature range, the shear storage modulus of composites is dependent upon the NaOH treatment of bamboo strips, while the temperature position of the viscoelastic transition is unmodified. The most important increase in storage modulus concerns the vitreous plateau. Composites with treated bamboo strips at 1% NaOH show higher storage modulus across the entire temperature range. For the bamboo strips treatments with 3 and 5%NaOH, considering error bars, any conclusion would be speculative.

### 3.4. Water Uptake Influence on Mechanical Properties

**Figure 8:**
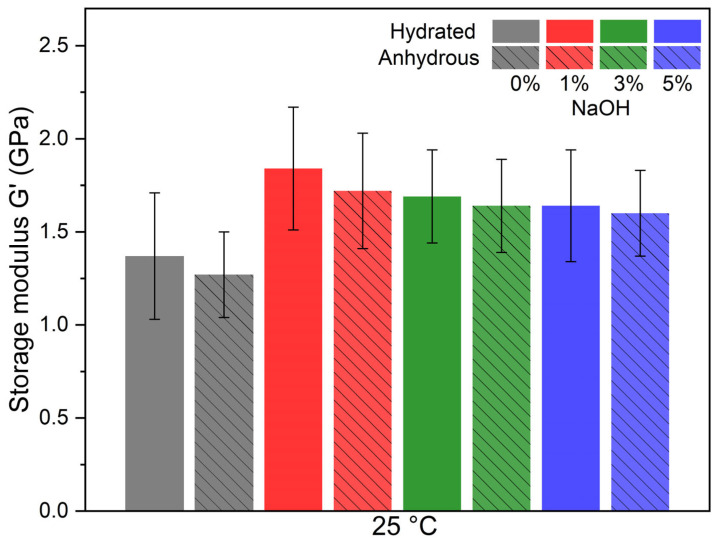
Conservative shear modulus at room temperature (25 °C) of bamboo strips/PA11-FR/composite. Grey bars: untreated bamboo strip composites; red bars: 1% NaOH treated composites; green bars: 3% NaOH treated composites; blue bars: 5% NaOH treated composites. Hydrated bamboo strips: unhatched bars; anhydrous bamboo strips: hatched bars.

Like all natural fibers, bamboo interacts with water, which could affect its mechanical properties at ambient temperature [39]. Therefore, samples were placed in a controlled atmosphere until weight stabilization. Then, two DMA ramps were performed, and the shear modulus was determined at room temperature for both hydrated and anhydrous samples. Figure 8 shows the shear modulus at room temperature of untreated and treated bamboo strips/PA11-FR composites, in the hydrated and anhydrous states. Taking into consideration error bars, it can be seen that mercerization treatment increases the shear modulus at room temperature. Moreover, there is no significant effect of water uptake at room temperature for the shear modulus of bamboo strips/PA11-FR composites.

Table 2 reports values of moisture content of bamboo strip/PA11-FR composites. Hydrated samples were placed in a controlled atmosphere at 65% RH; anhydrous samples are the same samples after two DMA runs: these values take into account bound water within the composite.

Composites obtained with untreated bamboo strips contain 3% less water compared to bamboo strips. For composites obtained with mercerized bamboo strips at different wt%, the water uptake is less than that of the untreated bamboo strips and the same as indicated on the TGA graph (Figure 3). PA11-FR has a barrier effect on water uptake with untreated bamboo strips, but this effect vanishes with treated bamboo strips. This evolution could be explained by the fast water absorption process favored by the mercerization treatment.

## 4. Conclusions

The effect of NaOH concentration during mercerization on the thermo-mechanical performances of bamboo strips and bamboo strip-reinforced PA11-FR composites was analyzed.

TGA analysis shows that mercerization treatment partially removes hemicellulose and low-molecular-weight lignin. High-molecular-weight lignin stabilized in temperature during the treatment. TGA thermograms recorded under synthetic air highlighted both physical structures of cellulose, i.e., crystalline and amorphous. The NaOH treatment removes amorphous cellulose without reducing the proportion of crystalline cellulose.

Tensile tests were performed on untreated and treated bamboo strips coming from different depths of the bamboo culm (0–400 µm and 400–800 nm). The Young modulus and ultimate strength remain uniform, with values around 9.7 GPa and 113 MPa, respectively, regardless of position, suggesting that bamboo strip extraction can be performed to a depth of 800 µm.

Bamboo strip-reinforced polymer composites were obtained by compression molding. DMA tests show that composites have a higher shear modulus (1.31 GPa at 25 °C) than the matrix across the entire temperature range (1.02 GPa at 25 °C). Composites containing bamboo strips treated with the solution at 1% NaOH have the highest shear modulus across the entire temperature range (1.72 GPa at 25 °C). Water uptake in composites, whether with treated or untreated bamboo strips, has no impact on the mechanical performance at room temperature. Further research involving sizing agents will be performed in order to reduce the water uptake of the composites.

## Figures and Tables

**Table 1:** Values of Young modulus (E) and ultimate strength (R_m_) of untreated (UT) and 1 wt% NaOH treated (T1) bamboo strips.

	UT-EXT	T1-EXT	UT-MID	T1-MID
E (GPa)	9.9 ± (1.4)	9.7 ± (2)	9.0 ± (1.2)	10.8 ± (1.8)
R_m_ (MPa)	108 ± (20)	113 ± (17)	98 ± (27)	124 ± (33)

**Table 2:** Moisture content (wt%) of untreated bamboo strips (UT-BS) and BS/PA11 FR composites.

	UT-BS	BS0/PA11FR	BS1/PA11FR	BS3/PA11FR	BS5/PA11FR
65% RH	8.4 ± 0.8	5.9 ± 0.3	7.4 ± 0.7	7.4 ± 0.6	6.5 ± 0.2
Anhydrous	0.2 ± 0.2	0.6 ± 0.3	1.3 ± 0.1	1.2 ± 0.9	1.1 ± 0.2

## Data Availability

Data are contained within the article.
